# PPARα and TCE: Keshava et al. Respond

**Published:** 2007-01

**Authors:** Nagalakshmi Keshava, Weihsueh A. Chiu, Jane C. Caldwell

**Affiliations:** U.S. Environmental Protection Agency, Washington, DC, E-mail: keshava.nagu@epa.gov

We appreciate the opportunity to discuss the issues raised by Klaunig et al. in their letter. First, we reiterate that, given the mini-monograph’s scope (Chiu et al. 2006), our article (Keshava and Caldwell 2006) was intended not to comprehensively review the role of peroxisome proliferator-activated receptor α (PPARα) agonism in trichloroethylene (TCE) toxicity but rather to “highlight some of the recently published literature on PPARα ... to help inform and illustrate the key scientific issues relevant to TCE risk assessment.” In addition, we considered not just hepatocarcinogenesis, but a broader range of modes of action (MOAs) and toxicity effects, necessitating a brief discussion of the article by Klaunig et al. (2003). Furthermore, because of the pending National Academy of Sciences report and revision of the TCE assessment, Klaunig et al.’s suggestion to examine whether the data “support or refute the role of PPARα in TCE-induced effects” would have been premature in the mini-monograph.

In their letter, Klaunig et al. state that there are “common and reproducible changes in gene expression associated with PPARα agonists.” However, as described by Klaunig et al. (2003), the well-characterized changes are largely peroxisomal or related to lipid metabolism, and thus not causally related to hepatocarcinogenesis. Hays et al. (2005) and the Federal Insecticide, Fungicide, and Rodenticide Act Science Advisory Panel [FIFRA SAP (2004)] suggested that the MOA underlying PPARα agonist-induced hepatocarcinogenesis has not been fully elucidated in that the specific target genes modulated by PPARα leading ultimately to liver cancer have not been identified. We share the concerns of Klaunig et al. about critically interpreting gene array data and the concerns of Voss et al. (2006) about also considering dose-, time course–, species-, and strain-related differences. Given reports that PPARα agonists have zonal differences in hepatocyte, peroxisomal, and mitochondrial proliferation, and in foci development (Anderson et al. 2004a; Bannasch 1996), zone-dependent and nonparenchymal cell responses (e.g., Kupffer cells) should also be taken into account. Finally, Table 2 of our article (Keshava and Caldwell 2006) illustrated the pleiotropic and varying liver responses of the PPARα receptor to various agonists, but we did not imply that these responses were responsible for carcinogenesis.

We agree with Klaunig et al. that PPARα-null mice have been useful in investigating the MOA for hepatocarcinogenesis, particularly for the strong agonist WY-14,643 {[4-chloro-6-(2,3-xylidino)-2-pyrimidinylthiol]acetic acid}. However, possible limitations of genetically modified mice, such as lack of complete tumor development or manipulation of the carcinogenic process, should be adequately characterized [U.S. Environmental Protection Agency) (EPA) 2005]. Maronpot et al. (2004) noted the need for lifetime studies to characterize background or spontaneous tumor patterns and life spans (including those of the background strain) for these models.

PPARα-null mice have baseline difference from wild-type mice that may render them more susceptible to toxic responses [e.g., reduced glycogen stores, altered responses to fasting, elevated plasma free fatty acids, fatty liver, impaired gluconeogenesis, significant hepatic insulin resistance (Lewitt et al. 2001)], or potentially shorten their life spans with chemical exposure (Anderson et al. 2004b; Hays et al. 2005) or with further genetic modification (Nohammer et al. 2003). A comparison of their life spans with those of background strains without treatment has not been reported. Moreover, in PPARα-null mice, Wheeler et al. (2003) reported alteration of cyclin-dependent kinase/cyclin complexes necessary for cell cycle progression and DNA synthesis, whereas Voss et al. (2006) found increased apoptosis and decreased mitosis with fumonisin treatment. Thus, the question remains whether PPARα-null mice may have different susceptibility to hepatocarcinogenesis not specific to the proposed PPARα MOA.

Furthermore, bioassay study designs need adequate sensitivity to detect carcinogenic responses or elucidate MOAs. Morimura et al. (2006) and Hays et al. (2005) used high concentrations (with mortality), few (and differing numbers of) animals in treated versus control groups, and differing periods of exposure (all ≤ 1 year) complicating study interpretation. Interestingly, in the “humanized” PPARα-null mouse after 44 weeks of treatment, Morimura et al. (2006) noted (along with decreased toxicity) a WY-14,643–induced adenoma resembling spontaneous tumors rather than those seen in PPARα agonist-treated wild-type mice; no tumors were observed in controls. This raises the question of whether, if tested for longer periods of time, the humanized mice might show significant responses with tumors more consistent with those induced by a variety of non-PPARα agonists and those observed in humans (Bannasch 1996; Su and Bannasch 2003).

We acknowledge the importance of Peters et al. (1997) demonstrating *in vivo* effects of WY-14,643 on replicative DNA synthesis– and hepatocarcinogenesis–involved PPARα activation. Furthermore, we agree that peroxisome proliferation per se is an associative rather than causal event in the MOA for hepatocarcinogenesis (described by Rusyn et al. 2000). However, Klaunig et al. (2003) proposed a “minimal set of data elements” to support their PPARα MOA in rodents that consists of “PPARα agonism combined with light- or electron-microscopic evidence of peroxisome proliferation” or other markers of peroxisome proliferation. In addition, Klaunig et al.’s claim that we (Keshava and Caldwell 2006) misconstrued their review (Klaunig et al. 2003) as focusing on DNA damage as a possible contributor to the MOA is incorrect; that hypothesis was discussed by Reddy and Rao (1989). We believe it is important to identify changes both specific to PPARα activation and related to carcinogenesis.

Voss et al. (2006) reported fumonisin-induced apoptosis, cell proliferation, gene changes, and liver lesions to be PPARα-independent but having some common target genes with PPARα agonists. Thus, we should not only understand a particular agent’s effects on the cell cycle and proliferation but also establish dependence on PPARα. Another issue is the applicability of the proposed MOA across PPARα agonists. Hays et al. (2005) noted that much of the literature on the PPARα MOA used WY-14,643, which induces sustained cell proliferation, whereas weaker agonists produce more transient responses (Marsman et al. 1988). Kraupp-Grasl et al. (1990) noted differences among agonists in their abilities to promote tumors and suggested that they should not necessarily be considered a uniform group. Finally, the discussion of the effects of PPARα agonists on mitochondrial function in our article (Keshava and Caldwell 2006) was intended to raise the issue for further investigation.

Similar issues with respect to PPARα have been discussed by recent scientific panels (FIFRA SAP 2004; U.S. EPA Science Advisory Board 2006). We believe that our article (Keshava and Caldwell 2006), Klaunig et al.’s letter, and this response help to further elucidate these complex issues for the assessment of TCE as well as other chemicals.
